# Coronavirus Disease 2019 (COVID-19) Crisis: Losing Our Immunity When We Need It the Most

**DOI:** 10.3390/biology10060545

**Published:** 2021-06-18

**Authors:** Abdelaziz Ghanemi, Mayumi Yoshioka, Jonny St-Amand

**Affiliations:** 1 Functional Genomics Laboratory, Endocrinology and Nephrology Axis, CHU de Québec-Université Laval Research Center, Québec, QC G1V 4G2, Canada; abdelaziz.ghanemi@crchudequebec.ulaval.ca (A.G.); mayumi.yoshioka@crchudequebec.ulaval.ca (M.Y.); 2 Department of Molecular Medicine, Faculty of Medicine, Laval University, Québec, QC G1V 0A6, Canada

**Keywords:** coronavirus disease 2019 (COVID-19), immunity

## Abstract

**Simple Summary:**

With the current coronavirus disease 2019 (COVID-19) crisis, humans have developed new habits and adapted to a novel socioeconomic reality. Indeed, measures including confinement and lockdown have led to mental health problems, economic crisis, and social isolation, among other consequences. These consequences, along with hand washing, sanitization, and face masks, would reduce our immunity against infections, including COVID-19. Such reduced immunity could impact not only our vulnerability to diseases but also the efficacy of vaccines that carry the biggest hope to putting an end to this COVID-19 pandemic. Thus, there is a need to review these approaches and optimize measures taken to limit the spread of COVID-19 by taking into consideration the possible impact of these measures on our immunity to fight COVID-19.

**Abstract:**

The ongoing coronavirus disease 2019 (COVID-19) crisis has led to a new socioeconomic reality with the acquisition of novel habits. Measures imposed by governments and health authorities such as confinement and lockdown have had important consequences, including mental health problems, economic crisis, and social isolation. Combined with newly acquired habits such as hand washing, sanitization, and face masks, these have all directly and indirectly led to reduced immunity. Such effects on the immune system not only impact the epidemiological profile with respect to COVID-19 and other infectious diseases but also limit the efficacy of the ongoing anti-COVID-19 vaccination campaign. Therefore, there is a need to review these approaches and optimize measures towards better population immunity, which is much needed during such an epidemic.

The ongoing coronavirus disease 2019 (COVID-19) crisis [1], as well as the measures imposed by governments, public health recommendations, the emergence of new variants, and the delay in vaccination campaigns, has put a greater spotlight on the importance of population immunity during the critical phase of this rapidly evolving pandemic. In this paper, we focus on the hypothesis that the measures taken during the pandemic and the socioeconomic situation caused by this crisis are reducing immunity at a period when we need it the most.

One of the most important risk factors for immunity problems is obesity, which negatively impacts immunity [2,3]. We believe that the measures imposed to limit the spread of COVID-19, including home confinement, curfew, closed gymnasiums, and work from home recommendations, result in sedentary behaviors [4], which would lead to an obesity pandemic [5]. As we have previously suggested, obesity also increases the vulnerability of patients recovering from COVID-19 as it can lead to an impaired regeneration homeostasis [6] and impact the development of immune cells.

The other measures characterizing this crisis are the periodic hand washing and sanitizing along with physical distancing. Both of these reduce our contact with different infections and pathogenic agents. Contact with pathogens, in a similar way to vaccines, stimulates the immune system [7,8] and increases its performance. However, with such limited interaction with the environment compared to that before the start of the COVID-19 crisis, less pathogens are detected by the immune system. This could limit its functional interaction as described by the hygiene hypothesis [9], especially within the context of the COVID-19 crisis [10] and the impact on the microbiome [11]. This is also of particular importance given the close link between the microbiome and immunity [12,13] as well as the link between microbiota composition and mental health, including anxiety and depression [14,15], during this COVID-19 outbreak as per the opinions of Venema et al. and Janda et al. and supported by the research of Yeoh et al. [16,17,18].

The hard economic situation caused by this COVID-19 crisis [19] has also made a contribution. For instance, many individuals have lost their jobs [20], and food insecurity has worsened [21] due to price inflation in the markets [22]. Therefore, the grocery options for individuals with limited income is more inclined towards unhealthy choices, including high-fat, high-sugar, and high-salt foods, which are more affordable but have an impact on immunity [23], including the microbiome [24,25], and further worsen obesity. In addition, the poor nutritive value of such an unhealthy diet limits the intake of nutrients like vitamins and therefore impacts the production of antibodies [26], among other immune functions [27]. In addition, diets also impact the microbiota [28,29] and therefore mental health and immunity.

Another consequence of the ongoing COVID-19 crisis is the impact on mental health [30,31] due to psychological stress, disturbed sleeping, and reduced social interaction, which can lead to problems including depression, anxiety, distress, and panic disorder [32,33,34]. These mental health issues during the COVID-19 crisis, along with the impact on immunity [35], could also increase the usage/intake of products such as drugs, alcohol, and tobacco [36,37] with consequent effect on immunity, as has been reported for alcohol consumption [38,39], tobacco [40,41], and drugs [42]. Within this context, it is worth highlighting the importance of pets as companions in reducing the discomfort caused by social isolation, with resulting benefits for both mental health (depression, anxiety, and stress) and physical activity [43], especially as they do not cause transmission of the COVID-19 virus [44].

Moreover, with the lockdown, some individuals may delay receiving the medical care they need or reduce the frequency of visits to these services [45,46], especially given most healthcare facilities would have reduced their regular services so that they could focus their energy on treating COVID-19 cases. This also has a significant impact on general health, including immunity.

## Conclusions

**Figure 1:**
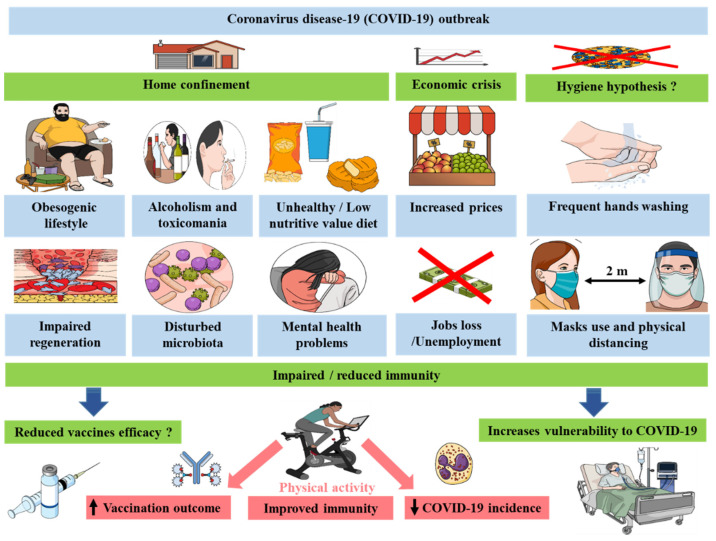
COVID-19 crisis measures lead to reduced immunity via diverse factors that could impact both COVID-19 morbidities and vaccines efficacy.

The consequences of this multifactorial reduced immunity (Figure 1) can not only increase the risk of developing a severe case of COVID-19 [47] and other infectious diseases [48,49] but also limit the efficacy of vaccines [50].

However, to what extent the effects on immunity due to the issues discussed in this paper (obesity, stress, mental health, hygiene hypothesis, etc.) impact the immune system compared to primary and secondary immunodeficiencies would depend on many factors, including the duration of exposure (which would be the period of the COVID-19 crisis), the severity of these factors, and individual physiological and pathological profiles. Other factors that could have an important influence include medication and the available medical care, which could worsen or improve the immunological outcome. Within this context, our theory indicates that these elements will have an immediate impact on immunity and vaccine effectiveness (pertinent to the current outcomes in this pandemic) as well as potential long-term effects that could persist in the future. Indeed, whereas factors such as disturbed microbiota and mental health problems [51] can be reversed following a healthy lifestyle and/or therapies, which will then improve immunity, other factors such as obesity can turn into a chronic status [52] with long-lasting consequences [53], including on immunity.

Therefore, it is urgent to find innovative solutions beyond home confinement and lockdown to get over this health crisis. Among the most efficient approaches would be to increase the physical activity of the population because exercise has been shown to improve immunity, including in the context of COVID-19 [54,55,56]. This could reduce the incidence of COVID-19 and improve vaccine efficacy. Importantly, the negative effects of this crisis on immunity described herein suggest the need to speed up the vaccination process before the effect of the vaccines is reduced, especially due to the emerging variants.

## Data Availability

Not applicable.
